# Which Parameter Related to Low-Density Lipoprotein Cholesterol is Superior for Predicting the Recurrence of Myocardial Infarction in Young Patients with Previous Coronary Heart Disease? A Real-World Study

**DOI:** 10.31083/RCM25721

**Published:** 2025-02-20

**Authors:** Feng Xu, Hao-Ran Xing, Hong-Xia Yang, Jin-Wen Wang, Xian-Tao Song, Hui-Juan Zuo

**Affiliations:** ^1^Department of Cardiology, Beijing Anzhen Hospital, Capital Medical University, 100029 Beijing, China; ^2^Department of Community Health Research, Beijing Institute of Heart Lung and Blood Vessel Diseases, Beijing Anzhen Hospital, Capital Medical University, 100029 Beijing, China

**Keywords:** coronary heart disease, LDL cholesterol, myocardial infarction, secondary prevention

## Abstract

**Background:**

Lowering low-density lipoprotein cholesterol (LDL-C) is a well-established strategy for the secondary prevention of coronary heart disease (CHD). However, the effectiveness of specific LDL-C parameters in predicting myocardial infarction (MI) recurrence in real-world settings remains inadequately explored. This study aims to examine the relationship between MI recurrence and various LDL-C parameters in young CHD patients.

**Methods:**

This retrospective cohort study involved 1013 patients aged 18–44 at the time of initial CHD diagnosis, collected from the cardiology department clinics at Beijing Anzhen Hospital between October 2022 and October 2023. LDL-C levels were assessed at the time of CHD diagnosis and at the final follow-up. The primary outcome was MI events, analyzed using survival analysis and logistic regression models to determine associations with LDL-C parameters.

**Results:**

The study included 1013 patients (mean age: 38.5 ± 3.9 years; 94.7% men), with a median follow-up time of 1.7 years. Initially, 13.6% had LDL-C levels <1.8 mmol/L, which increased to 37.8% by the study’s end. During follow-up, 96 patients (9.5%) experienced MI. While LDL-C <1.8 mmol/L at baseline showed a slightly lower cumulative incidence of MI than LDL-C ≥1.8 mmol/L, the difference was not statistically significant (log-rank *p* = 0.335). Reductions in LDL-C levels of ≥50% and the patterns of change did not correlate with decreased MI risk. However, LDL-C <1.4 mmol/L at the final measurement was associated with a reduced MI risk (adjusted odds ratio [OR]: 0.57, 95% confidence interval [CI]: 0.33–0.98) compared with LDL-C ≥2.6 mmol/L.

**Conclusions:**

This study suggests that the most important parameter related to LDL-C for predicting the recurrence of MI in young patients with a history of CHD is the ideal target LDL-C level. Lowering LDL-C to <1.4 mmol/L could potentially reduce MI risk, regardless of baseline LDL-C levels.

## 1. Introduction

Cardiovascular disease (CVD) is increasingly prevalent among younger individuals (aged 15–44), with the absolute number of CVD incidents and related deaths rising by 45.5% and 21.6%, respectively, from 1990 to 2019. Coronary heart disease (CHD) remains the leading cause of disability-adjusted life years (DALY) burden [1]. Previous studies have shown that individuals with a history of CHD are at an elevated risk for recurrent CHD events or cardiovascular-related hospitalizations across all age groups [2, 3, 4]. However, data on recurrent events in younger patients are scarce [5].

Lowering low-density lipoprotein cholesterol (LDL-C) is a crucial measure for both primary and secondary CHD prevention. Current evidence suggests that reducing LDL-C by >50% and managing other risk factors can lead to a decrease or even a disappearance of atherosclerotic plaques [6, 7, 8]. Continuous benefits have been observed in patients achieving LDL-C levels below 25 mg/dL [9, 10, 11]. Some clinical guidelines recommend lipid-lowering treatment with an LDL-C target of <1.8 mmol/L and a reduction of ≥50% from baseline for patients with CHD [12, 13]. The 2024 European Society of Cardiology (ESC) Guidelines for the management of dyslipidaemia and chronic coronary syndromes advocate for a more stringent LDL-C target of <1.4 mmol/L [14, 15]. Variations in LDL-C level changes over time are critical parameters that may indicate potential associations with CHD. However, the examination of dynamic trajectories in LDL-C levels remains underexplored in contemporary research [8]. Moreover, several studies have highlighted that elevated LDL-C levels at a young age correlate with increased CVD risks later in life, independent of subsequent adult exposures [16, 17, 18].

This real-world cohort study aimed to identify the LDL-C parameters that are most effective in predicting myocardial infarction (MI) recurrence in young patients with a history of CHD. We assessed the relationship between baseline LDL-C levels, LDL-C control at the end of the study, extent of LDL-C reduction, and patterns of LDL-C changes with subsequent MI events in patients aged 18–44 years at the time of their initial hospitalization for CHD.

## 2. Methods

### 2.1 Participants

This retrospective cohort study involved 1013 patients aged 18–44 years who were hospitalized for their first occurrence of CHD. Data were collected from the cardiology department at Beijing Anzhen Hospital between October 2022 and October 2023. Patients with secondary diagnoses of prior CHD, previous percutaneous coronary intervention (PCI), coronary artery bypass grafting (CABG), post-acute myocardial infarction (AMI) syndrome, chronic ischemic heart disease, heart failure (before or after CHD diagnosis), arteritis, congenital heart disease, and cancer, as well as those without specific information at the time of their first CHD diagnosis and those with a history of CHD <3 months prior were excluded.

The study protocol was reviewed and approved by the Ethics Committee of Beijing Anzhen Hospital. Written informed consent was obtained from all participants prior to their inclusion in the study.

### 2.2 Data Collection

Baseline information was collected from the hospital’s electronic medical records and other medical documents by trained abstractors. Collected demographic information included sex and age, while exposure to risk factors was assessed, including obesity, medical history of hypertension and diabetes, LDL-C levels, and smoking habits. Data on secondary prevention therapies, such as the use of aspirin, β-blockers, angiotensin receptor blockers/angiotensin-converting enzyme inhibitors (ARB/ACEI), and lipid-lowering therapy, were also gathered. Biochemical indicators recorded included hemoglobin A1c (HbA1c), triglycerides (TG), total cholesterol (TC), LDL-C, and high-density lipoprotein cholesterol (HDL-C). Coronary angiographic findings were reviewed to document the presence of occlusion or stenosis in the native coronary arteries (including the left main, left anterior descending, circumflex, and right coronary arteries). Follow-up information was collected through face-to-face interviews, which included changes in smoking habits, newly diagnosed hypertension and diabetes, secondary MI events, secondary preventive therapy, and coronary revascularization. For biochemical indicators, data from the last 3 months were recorded, or blood samples were collected and measured. Anthropometric data, such as height, weight, and blood pressure, were measured following standard procedures in the clinics.

### 2.3 Measurements and Diagnostic Criteria

Hypertension and diabetes were defined based on documented histories in medical records. Body mass index (BMI) was calculated as weight (kg) divided by the square of height (m^2^), with obesity defined as BMI ≥28 kg/m^2^. Smokers were defined as those who reported smoking >100 cigarettes or for >6 months, with a cessation time of <6 months. The optimal target for LDL-C was set at <1.8 mmol/L. Baseline LDL-C levels were categorized into two groups: <1.8 mmol/L and ≥1.8 mmol/L. The last LDL-C measurements were categorized into four groups: <1.4 mmol/L, 1.4–1.79 mmol/L, 1.8–2.59 mmol/L, and ≥2.6 mmol/L. The degree of LDL-C reduction was calculated as the difference between LDL-C levels at the end of the study and at baseline divided by the LDL-C levels at baseline. This reduction was categorized into four groups: <0%, 0%–29.9%, 30%–49.9%, and ≥50%. A trajectory model was used to determine the temporal development of LDL-C levels: LDL-C-uncontrolled (≥1.8 mmol/L at both baseline and end of the study), LDL-C-worsened (LDL-C <1.8 mmol/L at baseline and ≥1.8 mmol/L at the end), LDL-C-improved (LDL-C ≥1.8 mmol/L at baseline and <1.8 mmol/L at the end), and LDL-C-controlled (LDL-C <1.8 mmol/L at both baseline and end). Coronary artery disease was defined as the presence of significant stenosis (≥50%) in the main coronary arteries, as confirmed by coronary angiography, including the left main coronary artery (LM), left anterior descending artery (LAD), left circumflex artery (LCX), and right coronary artery (RCA). Single vessel lesion was defined as a stenosis of ≥50% in the LAD, LCX, or RCA. Multi-vessel disease is defined as a stenosis of ≥50% in two or three of these vessels (LAD, LCX, and RCA) or ≥50% in the LM.

The primary endpoint was secondary MI events. MI was defined according to established criteria [19], which included both ST-segment elevation MI (STEMI) and non-ST-segment elevation MI (NSTEMI), with information obtained from relevant hospital records.

### 2.4 Statistical Analysis

Categorical variables were expressed as counts and percentages, with differences in groups compared using chi-square tests or Fisher’s exact tests when any expected cell count was <5. Continuous variables with normal distributions were presented as mean ± standard deviation, while those with skewed distributions were presented as median (interquartile range). Student’s *t*-test was used to compare two independent samples for normally distributed continuous variables, and the Mann-Whitney U-test was used for continuous variables with skewed distributions. Kaplan-Meier survival analysis was utilized to compare the impact of baseline LDL-C levels on MI events, with the log-rank method employed to test for significant differences between groups. Logistic regression models were utilized to examine the relationships between the extent of LDL-C reduction, pattern of LDL-C changes, and last recorded LDL-C level with MI events. These models calculated crude and adjusted odds ratios (ORs) along with corresponding 95% confidence intervals (95% CIs). All reported *p*-values were two-sided, with *p* < 0.05 considered statistically significant. Statistical analysis was conducted using IBM SPSS Statistics version 25.0 (IBM, Armonk, NY, USA).

## 3. Results

### 3.1 Main Characteristics at Baseline and Study End

Our study included 1013 patients, with a mean age of 38.5 ± 10.8 years at the time of CHD diagnosis. The majority of participants were male (94.7%). Among these patients, 547 (54.0%) presented with multi-vessel disease, 786 (77.6%) underwent PCI, 155 (15.3%) were managed solely with medication, and 72 (7.1%) underwent CABG.

At baseline, the prevalence rates of hypertension (48.0% vs. 55.0%) and diabetes (19.6% vs. 26.7%) were lower than those at the end of the study. Conversely, the prevalence rates of smoking (75.1% vs. 36.2%) and obesity (37.9% vs. 34.2%) were higher at baseline. Among the patients who smoked at baseline, 51.8% (394 of 761) quit smoking during the study period. Significant improvements were observed in the mean levels of HbA1c, diastolic blood pressure (DBP), TC, LDL-C, HDL-C, non-HDL-C, and TG at the end of the study (all *p* < 0.05). Specifically, the mean LDL-C decreased from 2.83 ± 1.10 mmol/L to 2.28 ± 0.93 mmol/L, while the mean non-HDL-C decreased from 2.59 ± 1.10 mmol/L to 1.98 ± 1.07 mmol/L. A high percentage of patients received secondary preventive therapy, particularly by the end of the study. At baseline, 88.4% of patients were prescribed a statin, 8.5% were taking ezetimibe, and none were on a proprotein convertase subtilisin/kexin type 9 (PCSK9) inhibitor. By the end of the study, these percentages increased to 91.2%, 36.2%, and 3.8%, respectively (Table 1).

**Table 1.** **Main characteristics at baseline and end of the study**.

Parameters		Baseline	Study end	*p* value
Risk factors				
	Hypertension, n (%)	486 (48.0)	557 (55.0)	0.002
	Diabetes, n (%)	199 (19.6)	270 (26.7)	<0.001
	BMI ≥28, n (%)	384 (37.9)	346 (34.2)	0.079
	Current smoker, n (%)	761 (75.1)	367 (36.2)	<0.001
	HbA1c (%)	6.2 ± 1.3	6.1 ± 1.2	0.011
	SBP (mmHg, $\bar{x}$ ± s)	125 ± 17	124 ± 14	0.154
	DBP (mmHg, $\bar{x}$ ± s)	79 ± 12	78 ± 11	0.004
	TC (mmol/L, $\bar{x}$ ± s)	4.53 ± 1.41	3.90 ± 1.18	<0.001
	LDL-C (mmol/L, $\bar{x}$ ± s)	2.83 ± 1.10	2.28 ± 0.93	<0.001
	HDL-C (mmol/L, $\bar{x}$ ± s)	0.90 ± 0.21	0.94 ± 0.21	<0.001
	Non-HDL-C (mmol/L, $\bar{x}$ ± s)	2.59 ± 1.10	1.98 ± 1.07	<0.001
	TG (mmol/L, median [IQR])	1.93 (1.37, 2.81)	1.66 (1.15, 2.40)	<0.001
Drug use for secondary prevention				
	Aspirin, n (%)	942 (93.0)	964 (95.2)	0.038
	Beta receptor blocker, n (%)	772 (76.2)	855 (84.4)	<0.001
	ARB/ACEI, n (%)	604 (59.6)	735 (72.6)	<0.001
	Statin, n (%)	895 (88.4)	924 (91.2)	0.033
	Ezetimibe, n (%)	86 (8.5)	367 (36.2)	<0.001
	PCSK9 inhibitor, n (%)	0	38 (3.8)	<0.001

ARB/ACEI, angiotensin receptor blocker/angiotensin-converting enzyme inhibitor; BMI, body mass index; DBP, diastolic blood pressure; HbA1c, hemoglobin A1c; HDL-C, high-density lipoprotein cholesterol; IQR, interquartile range; LDL-C, low-density lipoprotein cholesterol; PCSK9, proprotein convertase subtilisin/kexin type 9; SBP, systolic blood pressure; TC, total cholesterol; TG, triglyceride.

### 3.2 Lipid Indicators across Different Years after Discharge

Lipid levels, including LDL-C, non-HDL-C, HDL-C, and TG, were assessed at various time points following discharge (Table 2). Compared with baseline, all lipid indicators showed significant improvements. The percentage of patients achieving ideal targets for LDL-C, non-HDL-C, and TG peaked in the first year post-discharge, followed by a subsequent decline (*p* < 0.05). In contrast, no significant differences in HDL-C levels were observed across the different years following discharge (*p* = 0.169). Initially, 4.6% of patients had LDL-C levels <1.4 mmol/L, while 13.6% had levels <1.8 mmol/L. These percentages increased to 17.2% and 37.8%, respectively, within the first year, before slightly decreasing to 12.5% and 28.6%, respectively, 3 years later.

**Table 2.** **Lipid levels across different years after discharge [n (%)]**.

Lipid parameters		Baseline (n = 1013)	Years from discharge				
			≤1 (n = 472)	1–3 (n = 349)	>3 (n = 192)	χ ^2^	*p* value
LDL-C group (mmol/L)						5.973	0.016
	<1.4	47 (4.6)	81 (17.2)	43 (12.3)	24 (12.5)		
	1.4–1.79	91 (9.0)	97 (20.6)	69 (19.8)	31 (16.1)		
	1.8–2.59	341 (33.7)	181 (38.3)	122 (35.0)	66 (34.4)		
	≥2.6	534 (52.7)	113 (23.9)	115 (33.0)	71 (37.0)		
HDL-C group (mmol/L)						1.888	0.169
	<1.0	747 (73.7)	302 (64.0)	229 (65.6)	134 (69.8)		
	≥1.0	266 (26.3)	170 (36.0)	120 (34.4)	58 (30.2)		
Non-HDL-C group (mmol/L)						27.440	<0.001
	<2.6	199 (19.6)	333 (49.4)	146 (41.8)	67 (34.9)		
	2.6–3.39	311 (30.7)	146 (30.9)	92 (26.4)	57 (29.7)		
	3.4–4.09	210 (20.7)	51 (10.8)	48 (13.8)	24 (12.5)		
	≥4.1	293 (28.9)	42 (8.9)	63 (18.1)	44 (22.9)		
TG group (mmol/L)						20.374	<0.001
	<1.7	409 (40.4)	281 (59.5)	165 (47.3)	81 (42.2)		
	1.7–2.29	225 (22.2)	86 (18.2)	75 (21.5)	44 (22.9)		
	≥2.3	379 (37.4)	105 (22.2)	109 (31.2)	67 (34.9)		

HDL-C, high-density lipoprotein cholesterol; LDL-C, low-density lipoprotein cholesterol; TG, triglyceride.

### 3.3 Parameters Related to LDL-C

At the study’s outset, 138 of 1013 individuals achieved the ideal LDL-C target of <1.8 mmol/L. By the end of the study, the proportion of individuals meeting this target significantly increased: 71.7% of those with baseline LDL-C <1.8 mmol/L compared with 28.1% of those with LDL-C ≥1.8 mmol/L at baseline (*p* < 0.001). The patterns of LDL-C changes were categorized as follows: LDL-C-uncontrolled (62.1%), LDL-C-worsen (3.8%), LDL-C-improved (24.3%), and LDL-C-controlled (9.8%).

We calculated the difference between baseline LDL-C and the endpoint levels, revealing the distribution of LDL-C reductions: 26.9% of patients experienced an increase in LDL-C (reduction <0%), 39.6% had a reduction of 0%–29.9%, 23.9% had a reduction of 30%–49.9%, and 9.6% had a reduction of ≥50%.

### 3.4 Impact of Baseline LDL-C Levels on the Occurrence of MI Events

**Fig. 1.**
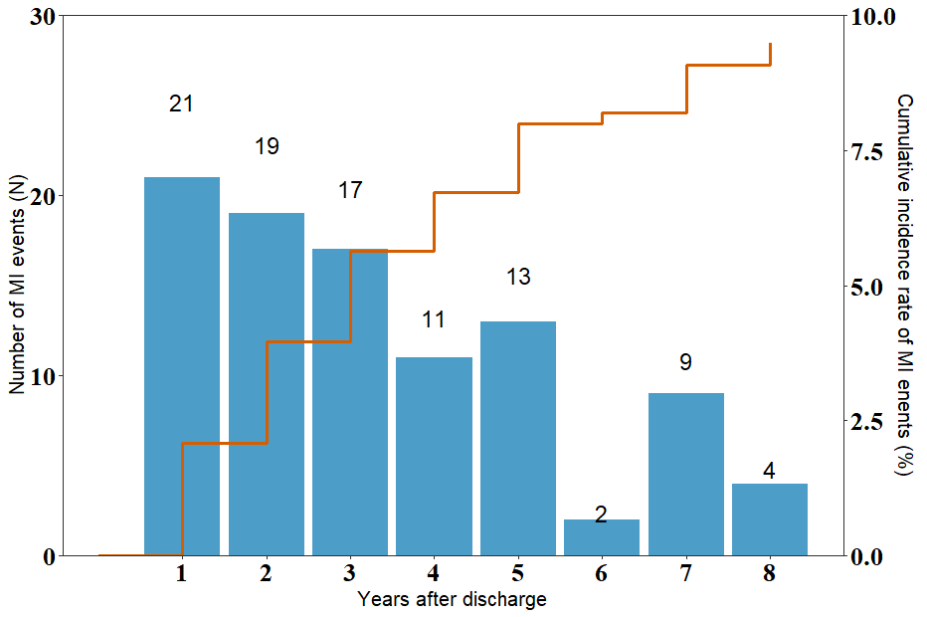
**Distribution of myocardial infarction (MI) events during a median follow-up period of 1.7 years**.

During a median follow-up period of 1.7 years (interquartile range: 1.0–3.0 years), 96 new cases of MI were identified. The crude rates of MI events over time were as follows: 1-year MI rate: 2.1%, 3-year rate: 5.8%, 5-year rate: 8.2%, and ≥8-year rate: 9.7% (Fig. 1).

**Fig. 2.**
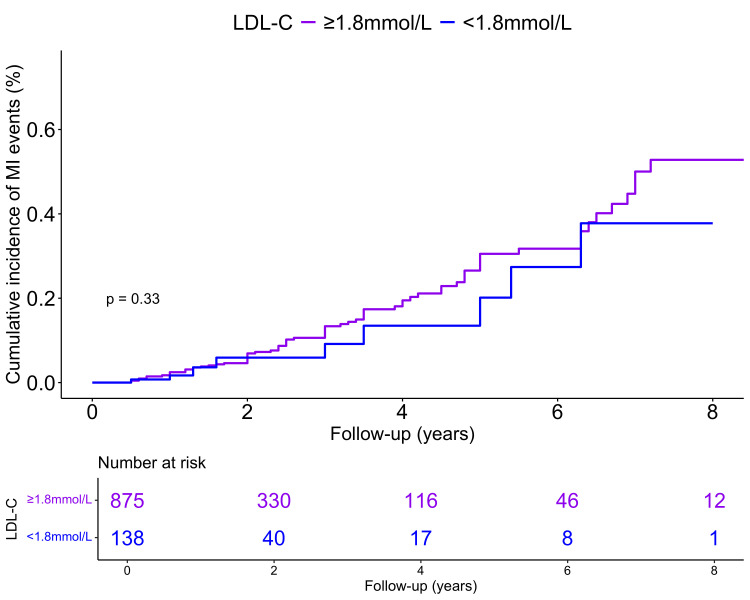
**Kaplan-Meier survival analysis curves for myocardial infarction (MI) events**. LDL-C, low-density lipoprotein cholesterol.

Kaplan-Meier survival analysis curves (Fig. 2) illustrated the cumulative incidence of MI events in two groups, based on a baseline LDL-C threshold of 1.8 mmol/L. The group with LDL-C levels <1.8 mmol/L exhibited a slightly lower cumulative incidence of MI events than those with LDL-C levels ≥1.8 mmol/L. However, this difference did not reach statistical significance (log-rank *p* = 0.335).

Univariate Cox regression analysis indicated no significant association between baseline LDL-C levels and the risk of MI occurrence (HR = 0.99, 95% CI: 0.84–1.17). After adjusting for age, sex, history of AMI, three-vessel disease, and treatment measures (Model 2), as well as for hypertension, diabetes, smoking status, HDL-C, TG, and BMI (Model 3), LDL-C levels remained insignificantly associated with MI risk (all *p* > 0.05). Sensitivity analyses using categorical variables showed that LDL-C levels <1.8 mmol/L did not significantly reduce the risk of MI compared with LDL-C levels ≥1.8 mmol/L, regardless of confounding adjustments (Table 3).

**Table 3.** **Cox regression analysis of the association between baseline LDL-C and MI events**.

Variable		Model 1			Model 2			Model 3		
		HR	95% CI	*p* value	HR	95% CI	*p* value	HR	95% CI	*p* value
Continuous LDL-C level		0.99	0.84–1.17	0.898	0.98	0.85–1.13	0.791	0.96	0.83–1.12	0.608
LDL-C group										
	≥1.8 mmol/L	ref			ref			ref		
	<1.8 mmol/L	0.72	0.35–1.42	0.340	0.63	0.36–1.09	0.100	0.64	0.36–1.12	0.119

Model 1: unadjusted; Model 2: adjusted for age, sex, history of AMI, three-vessel disease, and treatment measures; Model 3: adjusted for age, sex, history of AMI, three-vessel disease, treatment measures, hypertension, diabetes, current smoking, HDL-C, TG, and BMI at baseline. AMI, acute myocardial infarction; BMI, body mass index; CI, confidence interval; HDL-C, high-density lipoprotein cholesterol; HR, hazard ratio; LDL-C, low-density lipoprotein cholesterol; MI, myocardial infarction; TG, triglyceride.

### 3.5 Association between other Parameters Related to LDL-C and MI Occurrence

We analyzed the relationship between the pattern of LDL-C changes and the risk of MI events, using LDL-C-uncontrolled as the reference group. Both univariate and multivariate analyses failed to demonstrate a significant correlation between the trajectory of LDL-C changes (including LDL-C-worsen, LDL-C-improved, and LDL-C-controlled) and the occurrence of MI events (Table 4).

**Table 4.** **Association between other parameters related to LDL-C and the occurrence of MI**.

Changes in LDL-C			Model 1			Model 2			Model 3		
			OR	95% CI	*p* value	OR	95% CI	*p* value	OR	95% CI	*p* value
Pattern of LDL-C changes											
	LDL-C- uncontrolled		1			1			1		
	LDL-C-worsen		0.22	0.03–1.61	0.135	0.26	0.03–1.94	0.188	0.31	0.04–2.36	0.260
	LDL-C-improved		0.69	0.41–1.18	0.172	0.80	0.46–1.39	0.429	0.83	0.47–1.44	0.497
	LDL-C-controlled		0.73	0.34–1.56	0.411	0.84	0.38–1.87	0.672	0.93	0.41–2.09	0.853
Degree of LDL-C lowing (mmol/L)											
	Continuous LDL-C lowing		0.996	0.99–1.00	0.302	0.996	0.99–1.00	0.303	0.98	0.99–1.00	0.247
	Group										
		By 0–29.9%	1			1			1		
		By 30–49.9%	1.03	0.60–1.79	0.907	1.02	0.58–1.80	0.944	1.12	0.63–1.99	0.691
		By ≥50%	0.65	0.23–1.58	0.342	0.62	0.25–1.55	0.307	0.63	0.25–1.59	0.325
		By <0%	1.22	0.73–2.02	0.452	1.15	0.68–1.94	0.616	1.26	0.74–2.16	0.402
Last LDL-C measurement level (mmol/L)											
	Continuous LDL-C level		1.39	1.15–1.68	0.001	1.32	1.08–1.61	0.006	1.24	1.02–1.52	0.035
	Group										
		≥2.6	1			1			1		
		1.8–2.59	0.43	0.21–0.95	0.022	0.53	0.25–1.12	0.097	0.55	0.26–1.17	0.161
		1.4–1.79	0.56	0.31–1.02	0.057	0.71	0.39–1.32	0.283	0.78	0.41–1.47	0.437
		<1.4	0.45	0.27–0.75	0.002	0.53	0.32–0.92	0.023	0.57	0.33–0.98	0.043

Model 1: Unadjusted; Model 2: Adjusted for sex and age, history of MI, multi-vessel disease, treatment measures, and duration of CHD; Model 3: adjusted for sex and age, history of MI, multi-vessel disease, treatment measures, and duration of CHD, smoking status, BMI, hypertension, diabetes, TG, HDL-C at the end of the study. BMI, body mass index; CHD, coronary heart disease; CI, confidence interval; HDL-C, high-density lipoprotein cholesterol; LDL-C, low-density lipoprotein cholesterol; MI, myocardial infarction; OR, odds ratio; TG, triglyceride.

We also compared the risk of MI based on the degree of LDL-C level reduction, using a reduction of 0–29.9% as the reference group. The findings indicated that reductions of 30–49.9% or ≥50% were not associated with a decreased risk of MI events (Table 4).

Additionally, we examined the association between LDL-C levels at the last recorded measurement and MI risk, with LDL-C ≥2.6 mmol/L as the reference group. Results showed that individuals with LDL-C <1.4 mmol/L had a significantly decreased risk of MI events, with a crude OR of 0.45 (95% CI: 0.27–0.75) and an adjusted OR of 0.57 (95% CI: 0.33–0.98). However, no significant reductions in MI risk were observed among individuals with LDL-C levels of 1.8–2.59 mmol/L and 1.4–1.79 mmol/L (Table 4).

## 4. Discussion

Lowering LDL-C is a critical strategy for the secondary prevention of CHD. This study aimed to assess the association between various LDL-C parameters and MI incidence among young patients with a history of CHD. Our findings revealed no correlation between baseline LDL-C levels, extent of LDL-C reduction, or pattern of LDL-C changes and the risk of secondary MI events. However, individuals with LDL-C levels <1.4 mmol/L at the end of the study experienced a 43% reduced risk of secondary MI events compared with those with levels ≥2.6 mmol/L. These results suggest that maintaining LDL-C <1.4 mmol/L can effectively decrease the risk of secondary MI, regardless of initial LDL-C levels.

Previous studies have shown that elevated LDL-C levels at a young age may have a stronger association with CHD than lower LDL-C levels in adulthood [16, 17, 18]. This emphasizes the importance of baseline LDL-C levels before the onset of CHD. Moreover, increasing evidence suggests that reducing and maintaining LDL-C <1.8 mmol/L, alongside strict control of risk factors, can prevent both the onset and progression of atherosclerosis at younger ages [6, 7]. Additionally, significant benefits have been observed in patients achieving very low LDL-C levels (<25 mg/dL) [9, 10, 11]. Our findings indicated that LDL-C levels during admission were not associated with an elevated risk of secondary MI events among patients with CHD; however, lowering LDL-C levels can reduce this risk, with greater reductions leading to improved outcomes.

It is important to note that baseline LDL-C status does not represent time-varying exposure. During the follow-up period, LDL-C levels can be modified through interventions, such as LDL-C-lowering therapies and lifestyle changes [7]. Therefore, investigating the associations between dynamic changes in LDL-C status and the risks of secondary CHD events is essential [8]. Previous studies have examined the impact of dynamic changes in metabolic syndrome on CVD outcomes, finding that these changes can significantly affect long-term prognosis [20, 21]. In our study, participants were categorized into four groups based on LDL-C changes; however, the results indicated that these dynamic changes did not significantly alter the risks of MI events.

Clinical practice guidelines recommend aggressive LDL-C-lowering strategies with optimal targets, such as <1.8 mmol/L (70 mg/dL) and a 50% reduction for patients with very-high-risk atherosclerotic cardiovascular disease (ASCVD) [12, 13]. The 2019 guidelines from the European Society of Cardiology and the European Atherosclerosis Society suggest even stricter goals: targets of <1.4 mmol/L (<55 mg/dL) for very-high-risk patients or those with clinically evident ASCVD, and <1.0 mmol/L (<40 mg/dL) for very-high-risk patients who have experienced a secondary vascular event within the past 2 years [14, 15]. A study conducted on Japanese patients with CHD identified a potential threshold of 1.8 mmol/L for primary composite outcomes [22]. In our current study, we found that reducing LDL-C levels by ≥50% was not associated with a decreased risk of MI events. However, lowering LDL-C to <1.4 mmol/L appeared to decrease the risk of MI, irrespective of the baseline LDL-C level.

Despite improvements in guideline-recommended secondary prevention treatments, approximately 30% of patients with CHD achieved target reductions in LDL-C levels [2, 8, 23, 24, 25]. In China, only 13% of patients with very-high-risk ASCVD aged <45 years achieved the target LDL-C level of <1.4 mmol/L, whereas 24% of other patients with ASCVD reached the <1.8 mmol/L target [23]. In our study, only 17.2% of patients had LDL-C <1.4 mmol/L, and 37.8% had LDL-C <1.8 mmol/L. Several factors contribute to the challenges in meeting LDL-C goals. First, despite high rates of statin therapy, the use of high-intensity statins or maximally tolerated statin treatment may often be insufficient [11, 26, 27]. Second, the use of ezetimibe and PCSK9 inhibitors has been inadequate [28]. Finally, medication adherence is a common issue, with significant declines in the use of LDL-C-lowering drugs observed after discharge among patients with acute coronary syndrome [29]. Therefore, more effective lipid-lowering strategies should be developed, focusing on LDL-C levels prior to admission rather than those measured during hospitalization [30]. Early combination therapy, such as high-intensity statins combined with either ezetimibe or PCSK9 inhibitors, is recommended [14, 15, 31].

## 5. Limitations

This study has some potential limitations. First, it was conducted at a single center with a relatively small sample size, which may increase the risk of sampling bias and limit the generalizability of the findings. Second, the study included only patients who could provide hospital medical records for their initial CHD diagnosis and who consented to participate. This may introduce selection bias, potentially rendering the findings unrepresentative of all young patients with CHD. Third, we did not consider the type and dosage of statins and other medications used for LDL-C reduction, which limits our ability to assess the impact of different lipid-lowering strategies. Fourth, we did not collect data on lifestyle changes, such as dietary modifications and physical activity after discharge. These lifestyle factors can significantly influence cardiovascular outcomes and may serve as confounding variables when evaluating the impact of LDL-C-related indicators on MI. Finally, larger observational studies are needed to enhance the clinical implications of our findings.

## 6. Conclusions

In conclusion, this study suggests that the most important parameter related to LDL-C for predicting the recurrence of MI in young patients with a history of CHD is focusing on the ideal target LDL-C level. When LDL-C levels are reduced to sufficiently low levels, such as <1.4 mmol/L, the benefits in preventing the recurrence of MI significantly outweigh those of other parameters, including baseline LDL-C levels, extent of LDL-C reduction, and pattern of LDL-C changes.

## Availability of Data and Materials

The raw dataset analyzed in the current study are available from the corresponding author on reasonable request.

## References

[1] Zhang B, Luo L, Cai Y, Liu L, Ma X, Yang W (2024). Global burden of adolescent and young adult cardiovascular diseases and risk factors: Results from Global Burden of Disease Study 2019. *The Innovation Medicine*.

[2] Kerneis M, Cosentino F, Ferrari R, Georges JL, Kosmachova E, Laroche C (2022). Impact of chronic coronary syndromes on cardiovascular hospitalization and mortality: the ESC-EORP CICD-LT registry. *European Journal of Preventive Cardiology*.

[3] Peters SAE, Colantonio LD, Dai Y, Zhao H, Bittner V, Farkouh ME (2021). Trends in Recurrent Coronary Heart Disease After Myocardial Infarction Among US Women and Men Between 2008 and 2017. *Circulation*.

[4] Song J, Murugiah K, Hu S, Gao Y, Li X, Krumholz HM (2020). Incidence, predictors, and prognostic impact of recurrent acute myocardial infarction in China. *Heart (British Cardiac Society)*.

[5] Xu JJ, Jiang L, Song Y, Yao Y, Jia SD, Liu Y (2020). Related factors and the long-term outcome after percutaneous coronary intervention of premature acute myocardial infarction. *Zhonghua Xin Xue Guan Bing Za Zhi*.

[6] Mendieta G, Pocock S, Mass V, Moreno A, Owen R, García-Lunar I (2023). Determinants of Progression and Regression of Subclinical Atherosclerosis Over 6 Years. *Journal of the American College of Cardiology*.

[7] Devesa A, Ibanez B, Malick WA, Tinuoye EO, Bustamante J, Peyra C (2023). Primary Prevention of Subclinical Atherosclerosis in Young Adults: JACC Review Topic of the Week. *Journal of the American College of Cardiology*.

[8] Nielsen RV, Fuster V, Bundgaard H, Fuster JJ, Johri AM, Kofoed KF (2024). Personalized Intervention Based on Early Detection of Atherosclerosis: JACC State-of-the-Art Review. *Journal of the American College of Cardiology*.

[9] Wang N, Fulcher J, Abeysuriya N, Park L, Kumar S, Di Tanna GL (2020). Intensive LDL cholesterol-lowering treatment beyond current recommendations for the prevention of major vascular events: a systematic review and meta-analysis of randomised trials including 327 037 participants. *The Lancet. Diabetes & Endocrinology*.

[10] García RV, García JEP, Navas WD, Salmerón DM, Mateos DB (2022). Impact of a virtual lipid clinic on lipid-lowering therapy, LDL cholesterol levels, and outcomes in patients with acute coronary syndrome. *Journal of Clinical Lipidology*.

[11] Underberg J, Toth PP, Rodriguez F (2022). LDL-C target attainment in secondary prevention of ASCVD in the United States: barriers, consequences of nonachievement, and strategies to reach goals. *Postgraduate Medicine*.

[12] Grundy SM, Stone NJ, Bailey AL, Beam C, Birtcher KK, Blumenthal RS (2019). 2018 AHA/ACC/AACVPR/AAPA/ABC/ACPM/ADA/AGS/APhA/ASPC/NLA/PCNA Guideline on the Management of Blood Cholesterol: A Report of the American College of Cardiology/American Heart Association Task Force on Clinical Practice Guidelines. *Journal of the American College of Cardiology*.

[13] Joint Committee on the Chinese Guidelines for Lipid Management (2023). Chinese guidelines for lipid management (2023). *Zhonghua Xin Xue Guan Bing Za Zhi*.

[14] Mach F, Baigent C, Catapano AL, Koskinas KC, Casula M, Badimon L (2020). 2019 ESC/EAS Guidelines for the management of dyslipidaemias: lipid modification to reduce cardiovascular risk. *European Heart Journal*.

[15] Vrints C, Andreotti F, Koskinas KC, Rossello X, Adamo M, Ainslie J (2024). ESC Guidelines for the management of chronic coronary syndromes: Developed by the task force for the management of chronic coronary syndromes of the European Society of Cardiology (ESC) Endorsed by the European Association for Cardio-Thoracic Surgery (EACTS). *European Heart Journal*.

[16] Zhang Y, Vittinghoff E, Pletcher MJ, Allen NB, Zeki Al Hazzouri A, Yaffe K (2019). Associations of Blood Pressure and Cholesterol Levels During Young Adulthood with Later Cardiovascular Events. *Journal of the American College of Cardiology*.

[17] Zhang Y, Pletcher MJ, Vittinghoff E, Clemons AM, Jacobs DR, Allen NB (2021). Association Between Cumulative Low-Density Lipoprotein Cholesterol Exposure During Young Adulthood and Middle Age and Risk of Cardiovascular Events. *JAMA Cardiology*.

[18] Domanski MJ, Tian X, Wu CO, Reis JP, Dey AK, Gu Y (2020). Time Course of LDL Cholesterol Exposure and Cardiovascular Disease Event Risk. *Journal of the American College of Cardiology*.

[19] Thygesen K, Alpert JS, Jaffe AS, Chaitman BR, Bax JJ, Morrow DA (2019). Fourth universal definition of myocardial infarction (2018). *European Heart Journal*.

[20] Park S, Lee S, Kim Y, Lee Y, Kang MW, Han K (2019). Altered Risk for Cardiovascular Events with Changes in the Metabolic Syndrome Status: A Nationwide Population-Based Study of Approximately 10 Million Persons. *Annals of Internal Medicine*.

[21] He D, Zhang X, Chen S, Dai C, Wu Q, Zhou Y (2021). Dynamic Changes of Metabolic Syndrome Alter the Risks of Cardiovascular Diseases and All-Cause Mortality: Evidence from a Prospective Cohort Study. *Frontiers in Cardiovascular Medicine*.

[22] Sakuma M, Iimuro S, Shinozaki T, Kimura T, Nakagawa Y, Ozaki Y (2022). Optimal target of LDL cholesterol level for statin treatment: challenges to monotonic relationship with cardiovascular events. *BMC Medicine*.

[23] Li S, Liu HH, Guo YL, Zhu CG, Wu NQ, Xu RX (2021). Improvement of evaluation in Chinese patients with atherosclerotic cardiovascular disease using the very-high-risk refinement: a population-based study. *The Lancet Regional Health*.

[24] Shah NP, Page C, Green CL, Gao M, Cavalier J, McGarrah RW (2023). Bending the Cardiovascular Event Curve by Evaluating the Potential Impact of Achieving Low-Density Lipoprotein Cholesterol Goal Across a Large Health System Among Secondary Prevention Patients. *The American Journal of Cardiology*.

[25] März W, Dippel FW, Theobald K, Gorcyca K, Iorga ŞR, Ansell D (2018). Utilization of lipid-modifying therapy and low-density lipoprotein cholesterol goal attainment in patients at high and very-high cardiovascular risk: Real-world evidence from Germany. *Atherosclerosis*.

[26] Koskinas KC, Siontis GCM, Piccolo R, Mavridis D, Räber L, Mach F (2018). Effect of statins and non-statin LDL-lowering medications on cardiovascular outcomes in secondary prevention: a meta-analysis of randomized trials. *European Heart Journal*.

[27] Mahtta D, Ramsey DJ, Al Rifai M, Nasir K, Samad Z, Aguilar D (2020). Evaluation of Aspirin and Statin Therapy Use and Adherence in Patients with Premature Atherosclerotic Cardiovascular Disease. *JAMA Network Open*.

[28] Martin GJ, Teklu M, Mandieka E, Feinglass J (2022). Low-Density Lipoprotein Cholesterol Levels in Coronary Artery Disease Patients: Opportunities for Improvement. *Cardiology Research and Practice*.

[29] Atkins ER, Du X, Wu Y, Gao R, Patel A, Chow CK (2017). Use of cardiovascular prevention treatments after acute coronary syndrome in China and associated factors. *International Journal of Cardiology*.

[30] Marcos-Garcés V, Merenciano-González H, Martínez Mas ML, Palau P, Climent Alberola JI, Perez N (2023). Short-Course High-Intensity Statin Treatment during Admission for Myocardial Infarction and LDL-Cholesterol Reduction-Impact on Tailored Lipid-Lowering Therapy at Discharge. *Journal of Clinical Medicine*.

[31] Makhmudova U, Samadifar B, Maloku A, Haxhikadrija P, Geiling JA, Römer R (2023). Intensive lipid-lowering therapy for early achievement of guideline-recommended LDL-cholesterol levels in patients with ST-elevation myocardial infarction (“Jena auf Ziel”). *Clinical Research in Cardiology: Official Journal of the German Cardiac Society*.

